# Miscellaneous Medical Intelligence

**Published:** 1850-11

**Authors:** 


### ARTICLE IX.

### MISCELLANEOUS MEDICAL INTELLIGENCE.

The Transylvania University at Lexington, Ky., the oldest medical school in the West, and one of the most respectable, has reduced the price of its Professors' Tickets from fifteen to ten dollars each.

Dr. Blatchford, of Troy, N. Y., recently induced premature labor. to prevent the necessity of Craniotomy, by M. Cohen's method of injecting tar water into the Uterus, which proved successful.

Dr. Drake has been re-appointed to the Chair of Practice in the University of Louisville.

Dr. Baxley, of Baltimore, Md., has been appointed to the Chair of Anatomy in the Medical College of Ohio, in place of Dr. Shortwell, deceased.

Dr E. H. Davis, of Chilecotha, O., has been appointed Professor of Nat. Med. in the New York Medical College.

Prof. Mott, of New York, has been elected Emeritus Professor of Operative Surgery and Surgical Anatomy in the College of Physicians, (old school) of New York.

Prof. Peaslee has removed an ovarian tumor weighing 24 lbs.

The Ohio Weslyan University proposes to organize a medical department to be located in Cincinnati, as we learn by the newspapers.

Medical Cliques in New York, it is said in the Medical Gazette, combine to call *each other only* in consultations.

The prospects of the Kentucky School of Medicine are said to be of the most flattering character. A very commodious building, well adapted to its wants, has been secured through the liberality of two citizens of Louisville.

Prof. Wm. B. Herrick has been appointed by the U. S. authorities Surgeon to the Chicago Marine Hospital to take effect when the Hospital opens.

